# Reliability Analysis of Vertebral Landmark Labelling on Lumbar Spine X-ray Images

**DOI:** 10.3390/diagnostics13081411

**Published:** 2023-04-13

**Authors:** Jun-Su Jang, Joong Il Kim, Boncho Ku, Jin-Hyun Lee

**Affiliations:** 1 Digital Health Research Division, Korea Institute of Oriental Medicine, 1672 Yuseong-daero, Yuseong-gu, Daejeon 34054, Republic of Korea; 2 Institute for Integrative Medicine, Catholic Kwandong University International St. Mary’s Hospital, 25 Simgok-ro 100 beon-gil, Seo-gu, Incheon 22711, Republic of Korea

**Keywords:** inter-rater reliability, vertebral landmarks, lumbar spine, X-ray, quantitative analysis

## Abstract

Vertebral landmark labelling on X-ray images is important for objective and quantitative diagnosis. Most studies related to the reliability of labelling focus on the Cobb angle, and it is difficult to find studies describing landmark point locations. Since points are the most fundamental geometric feature that can generate lines and angles, the assessment of landmark point locations is essential. The aim of this study is to provide a reliability analysis of landmark points and vertebral endplate lines with a large number of lumbar spine X-ray images. A total of 1000 pairs of anteroposterior and lateral view lumbar spine images were prepared, and 12 manual medicine experts participated in the labelling process as raters. A standard operating procedure (SOP) was proposed by consensus of the raters based on manual medicine and provided guidelines for reducing sources of error in landmark labelling. High intraclass correlation coefficients ranging from 0.934 to 0.991 verified the reliability of the labelling process using the proposed SOP. We also presented means and standard deviations of measurement errors, which could be a valuable reference for evaluating both automated landmark detection algorithms and manual labelling by experts.

## 1. Introduction

Abnormal alignment of the spine itself is a diagnostic criterion for several spinal diseases, such as scoliosis and spondylolisthesis [1,2], and it is also a major factor of low back pain and spinal instability [3,4]. These pathological conditions of the spine are usually diagnosed through radiography (especially X-ray images) because it can quickly acquire the pathological condition and location information of the bone, has low radiation exposure, and has wide availability [5,6]. Recently, several methods for diagnosing spinal misalignment based on medical images, such as ultrasound, CT, and MRI, have been developed, but these are extensions of X-ray-based measurement, which is still considered the gold standard [7,8,9,10].

In the field of manual medicine, such as chiropractic, osteopathy, and Chuna manual therapy, abnormalities of vertebral alignment are defined as subluxation, malformation, or dysfunction, mainly using diagnostic methods such as visual observation, palpation, or X-ray [11,12,13,14]. In Chuna manual therapy [15,16,17], vertebral alignment is evaluated according to the relative positions of the upper and lower vertebral bodies [18]. Among many diagnostic methods, X-ray has the advantage of being objective and highly reproducible [19].

For objective and quantitative diagnosis, it is necessary to define anatomical landmark points such as a central point of the vertebral body or the four outer corner points of the two-dimensional planar shape of the vertebra projected in X-ray image [20,21]. Since points are the most fundamental geometric features, they are commonly used not only in medical images but also in other fields, such as face and human body analyses [22,23]. Points can be used to generate the other landmarks, including lines and polygons, and useful measurements such as lengths, angles, curvatures and areas can be calculated based on the point locations. Therefore, evaluating the accuracy of landmark point locations is essential to quantitative analysis. For example, landmark points are used to define the upper/lower endplate lines and segmentation region of a vertebral body in spine images. One of the most popular quantitative spinal landmark analyses is vertebral angle assessment, including the Cobb angle [24]. Many previous studies [24,25,26,27,28,29,30,31] have reported the inter- and intrarater reliability of angle assessment by means of intraclass correlation coefficients [32]. Cobb angle assessment is the most popular application in this field; however, it is difficult to find studies utilizing landmark point locations.

In this study, we conducted landmark labelling experiments on lumbar spine (L-spine) X-ray images. A detailed definition of landmark points of the vertebral body is essential to reduce the landmark point location error among experts, i.e., raters. To establish a standard operating procedure (SOP) for landmark labelling of the vertebral bodies, a group of 12 manual medicine experts participated in this study. In previous morphometry studies [33,34], landmarks were described using qualitative sentences and illustrated examples. Based on such qualitative descriptions, landmark positions were measured quantitatively. While utilizing the previous morphometry studies, we prepared a SOP from the perspective of Chuna manual medicine. This SOP was proposed by consensus of the group of experts based on prior literature and provides guidelines for reducing sources of error in landmark labelling. Through this process, this study aims to present the inter-rater reliability for the definition of four landmark points and upper/lower endplate lines in anteroposterior (AP) and lateral (LAT) views of L-spine X-ray images. We also present the means and standard deviations of the labelling variation among raters. The findings can be cited as a criterion for evaluating the performance of an automated landmark detection algorithm.

## 2. Materials and Methods

### 2.1. Research Ethics Approval

This trial received complete ethical approval from the Ethics Committee of Catholic Kwandong University International St. Mary’s Hospital on 16 September 2019 (document number: 19YOEN IRB028, assignment number: IS19OSSE0031).

### 2.2. Inclusion and Exclusion Criteria of X-ray Images in the Study

The X-ray images of this study were collected through retrospective chart analysis through the following inclusion and exclusion criteria. Initially, L-spine X-ray for 2000 patients (AP view 2000 cases, LAT view 2000 cases) was collected. Among the collected cases, those of 1000 patients were finally included. All X-ray image files used in the study were extracted through an anonymization process that excluded personally identifiable information.

#### 2.2.1. Inclusion Criteria

(a) Male or female sex and age 18–80 years;
(b) L-spine X-ray performed at the Neurosurgery of Catholic Kwandong University International St. Mary’s Hospital from February 2014 to May 2019.

#### 2.2.2. Exclusion Criteria

(a) History of lumbar surgery for inducing structural changes in the lumbar vertebrae (e.g., fracture and screw fixation);
(b) Severe lumbar degenerative changes or structural abnormalities of the lumbar spine;
(c) Low quality of X-ray image, which was considered unsuitable for labelling.

**Figure 1:**
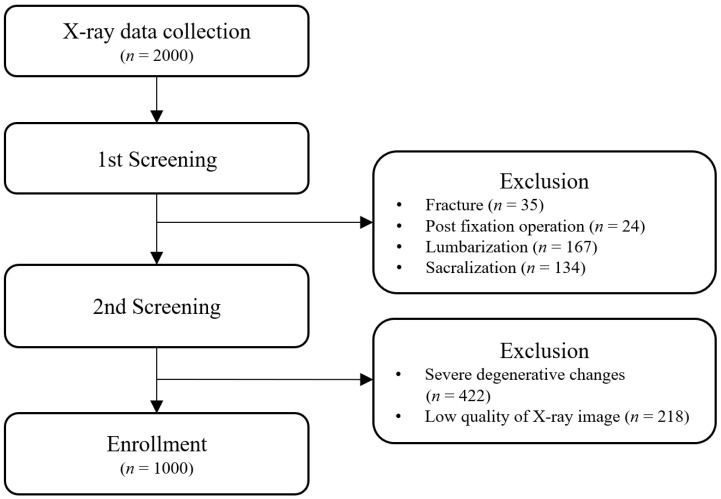
Flow diagram indicating detailed exclusion steps.

Figure 1 shows the detailed exclusion steps in data collection.

### 2.3. Rater Groups for Landmark Labelling

Lumbar X-ray labelling was performed by 12 Korean medical doctors (KMDs) who satisfied at least one of the following criteria: (1) a specialist with certification from Korean Medicine Rehabilitation; (2) a certified lecturer or educational board member of the Korean Society of Chuna Manual Medicine for Spine and Nerves (KSCMMSN); and (3) an individual with a published study regarding spinal alignment diagnosis. The 12 selected raters were divided into 2 groups of 6 raters each to conduct the study.

The two rater groups performed vertebral landmark labelling according to the SOP. A total of 1000 patient cases were labelled. Since the number of cases was large and labelling is labour intensive, total patient cases were divided into 2 sets of 600 patient cases for each group. Specifically, 400 patient cases in each group and 200 common patient cases in both groups were prepared (cases 1 to 600 were assigned to Group A, cases 401 to 1000 were assigned to Group B, and cases 401 to 600 were assigned to both groups). For AP and LAT views, the raters labelled 4 landmark points for each lumbar vertebra (20 points for L1-L5 vertebrae in total).

### 2.4. SOP for Landmark Labelling

**Figure 2:**
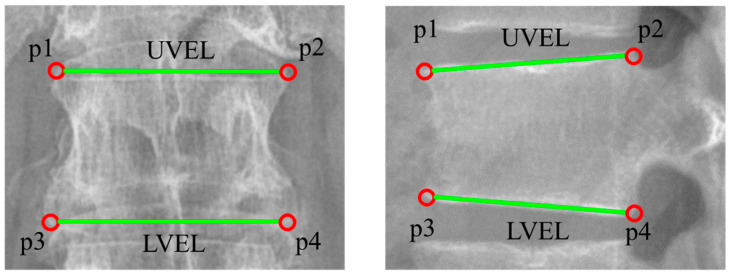
Definition of landmark points and upper and lower vertebral endplate lines ((**Left**): AP view, (**Right**): LAT view).

The landmarks of the vertebral body were defined based on previous studies [14,18] and the Chuna manual therapy textbook commonly used in Korean medical colleges [35]. Landmarks for each vertebra were defined as four points (p1: top left, p2: top right, p3: bottom left, p4: bottom right). The lines created by connecting the upper and lower landmark points represent the upper vertebral endplate line (UVEL) and lower vertebral endplate line (LVEL), respectively (Figure 2).

The four landmark points for each vertebra were marked according to the following 4 principles. When some principles conflicted, we gave priority to the ones listed higher.

(a) The UVEL and LVEL must be parallel to the upper and lower vertebral endplates, respectively.
(b) The landmark points must be marked on the vertebral body margins (border lines). When the margins appear as two lines due to rotation of the vertebra, the landmark points should be marked on the outer line.
(c) Lines created by connecting landmark points vertically must be parallel and close to the outer margin of the vertebral body; this would be helpful in the evaluation of laterolisthesis in AP view images and of anterolisthesis and retrolisthesis in LAT view images. For the evaluation of anterolisthesis and retrolisthesis in LAT view images, landmark points should be located close to the anterior vertebral line and the posterior vertebral line.
(d) If variations due to osteophytes and degenerative changes are observed, they can be ignored. Assuming that the vertebral body is an ideal cylinder shape, the UVEL and LVEL should be close to the diameter of the cylinder bases (top and bottom circles). If the margins of upper and lower vertebral endplates are convex, the UVEL and LVEL can be seen as penetrating the vertebral body. If the margins of the upper and lower vertebral endplates are concave, the UVEL and LVEL can be seen outside the vertebral body.

### 2.5. Statistical Analysis

The reliability of the landmark points labelled by the raters on each vertebra on both AP and LAT views for each patient’s X-ray image was evaluated in two aspects: the locations of the labelled points and the angles of the UVEL and LVEL. To obtain the variability in the landmark points among the raters, the Euclidean distances from the mean location of the raters’ labelled points for each vertebral body in each image were calculated. The angles of the UVEL and LVEL at each vertebral body were also obtained to assess the rater’s agreement. For the angles of the UVEL and LVEL, each raters’ root mean-square error (RMSE) was calculated to quantify the raters’ error of measurement in terms of the shape of the vertebral body.

Both the Euclidean distances of the labelled points and the RMSEs of the angles of the UVEL and LVEL were summarized as the mean and standard deviation (SD) for the cases in each of the two rater groups and for cases rated by both groups. To identify the homogeneity between the two rater groups in terms of variability, a linear mixed effects model (LMM) was performed to assess the difference in the Euclidean distances and the RMSEs of the angles between groups. The inter-rater reliability according to the measures was evaluated using the intraclass correlation coefficient (ICC) (two-way random-effects model, absolute agreement). The 95% confidence interval (CI) of the estimated ICCs was estimated based on 500 bootstrapped distributions. An ICC value can be interpreted as poor (ICC < 0.50), moderate (0.50 ≤ ICC < 0.75), good (0.75 ≤ ICC < 0.90), and excellent (ICC ≥ 0.90) [32]. All data preprocessing and statistical analyses were conducted using the statistical software R (version 4.1.2, released 1 November 2021).

The image resolution was 1953 × 3000 and one pixel was mapped to 0.143 mm. The landmark point locations were converted from image pixel to mm for our distance analysis.

## 3. Results

The mean variations in the landmark points at each lumbar vertebra (L1–L5) of AP and LAT views ranged from 1.397 mm to 3.606 mm and from 1.047 mm to 1.617 mm, respectively, for each rater group (Table 1). The variations due to the rater group were not statistically significant for all landmark points for both AP and LAT views (Table 1). The inter-rater reliability for all landmark points on AP and LAT views labelled by both rater groups showed excellent ICCs from 0.935 to 0.996 (Table 1). When compared to the landmark points labelled on the other vertebrae, the landmark points for L5 on the AP view image showed lower reliability for both rater groups in terms of the measured errors (2.492 mm to 3.606 mm) and ICCs (0.935 to 0.970).

For cases labelled by all 12 raters, the mean variations in the landmark points ranged from 1.185 mm to 3.430 mm on AP and LAT views (Table 2). The inter-rater reliability of the landmark points labelled by all 12 raters also showed excellent ICCs from 0.934 to 0.991 (Table 2). Similar to the results of the two rater groups, the landmark points of L5 on the AP view rated by all 12 raters showed lower inter-rater reliability compared to the other vertebrae. The measured errors ranged from 2.677 mm to 3.430 mm, and the ICCs ranged from 0.934 to 0.967. We also performed the detailed analysis in each of the x and y directions, separately. Instead of the Euclidean distance, distances in x and y directions were analysed. The mean variations and inter-rater reliability in the x and y directions are shown in Tables S1–S3 (Supplementary Material).

The variation in the RMSEs of the angles of the UVEL and LVEL ranged from 0.572° to 1.042° on the AP view and from 0.918° to 1.532° on the LAT view for both rater groups (Table 3). There were no significant differences between the two rater groups in terms of the angles of the UVEL and LVEL for any vertebra for either the AP or LAT views (Table 3). The inter-rater reliability for the angles of the UVEL and LVEL for all vertebrae varied from a good ICC of 0.759 to an excellent ICC of 0.942 for the AP view and from an ICC of 0.938 to 0.969 (all excellent) for the LAT view (Table 3).

The variations in the RMSEs for the angles of the UVEL and LVEL for the cases rated by all 12 raters ranged from 0.577° to 0.979° on the AP view and from 0.906° to 1.486° on the LAT view (Table 4). The inter-rater reliability of the angles of the UVEL and LVEL assessed by all 12 raters ranged from a good ICC of 0.801 to an excellent ICC of 0.942 for the AP view and showed excellent ICCs ranging from 0.942 to 0.963 for the LAT view. Similar to the tendency shown in the result for the landmark points, the L5 vertebra on both AP and LAT views shows higher variability and lower reliability than the other vertebrae in both results evaluated by each rater group and by all 12 raters.

The boxplots of our results are shown in Figures S1–S3. Additionally, we investigated the effects of patient age and sex on the mean variations of angles on UVEL and LVEL, as well as the mean variations of the landmark points labelled by the raters, and these results are presented in Tables S4 and S5. Our omnibus tests results showed significant variations due to age and sex. Although we found significant variations due to age and sex, we treated them as random errors since our primary focus was to quantify the rater’s variability in terms of the assessment of landmark points on X-ray images.

## 4. Discussion

Manual medicine is a field of complementary and alternative medicine. Clinical studies on manual medicine, including Chuna manual medicine, chiropractic, and osteopathy, often encounter challenges with inter-rater reliability due to a heavy dependence on imprecise palpation-based diagnostic techniques [36,37,38]. To overcome these limitations and ensure greater ease of evaluation in clinical settings, X-ray imaging is the most widely used method for the quantitative analysis of spinal structures in manual medicine.

While X-ray evaluation is advantageous for Chuna manual medicine, achieving a good quantitative diagnosis requires clear landmark locations with high inter-rater reliability. We performed a comprehensive analysis of Chuna medicine textbooks and morphometry studies to determine such landmarks. Subsequently, we derived a detailed SOP to locate the four landmark points for a vertebra. However, since the vertebral body has a complex shape, it is difficult to define landmark point locations that all raters can label as the same pixel in a given image. The definition of the landmark point location is presented in human languages and pictures; therefore, variability among raters is unavoidable.

Because the vertebral body is not an ideal cylinder and its projected shape on a 2D image is not rectangular, it possesses no mathematically true corner points. Human experts can approximate the projected shape of the vertebral body as a rectangle and assign landmark points close to the imaginary corner points of the rectangle. The SOP should help this process to maintain high reliability among raters. For this reason, we prepared 4 principles for determining the landmark point locations in the SOP.

To establish the ground truth for each landmark point location in each image, the mean location of the raters’ labelled points was calculated. Then, the Euclidean distance from the mean location to each rater’s labelled point location was calculated to obtain the mean and SD for the Euclidean distance for each landmark point. The ICC results (Table 1, Table 2, Table 3 and Table 4) show that the proposed SOP yielded good to excellent reliability. For all vertebrae except L5, the ICCs were higher than 0.9, which is interpreted as excellent reliability. For L5 on the AP view, the ICC for L5_UVEL was 0.839, and the ICC for L5_LVEL was 0.801 (Table 4), both lower than the ICCs of other vertebrae but still in the good range of 0.75 ≤ ICC < 0.90 [33]. Since the angles of the UVEL and LVEL were derived from the locations of a pair of landmark points, the mean value for the Euclidean distance of each point of L5 was also higher than that of the other points. The L5 region is relatively difficult to label because it is superposed by L4 or the sacrum on the AP view.

In L-spine images, unlike in whole spine images, there can be different opinions on the L1 locations among the raters. The L1 locations may be determined as those corresponding to L2 or T12 (the last thoracic vertebra) due to sacralization or lumbarization. Although sacralization and lumbarization were excluded during the screening process, there were cases where some raters evaluated the presence of sacralization and lumbarization, depending on their interpretation. These cases were regarded as missing values in our study, since we focused on the reliability of landmark point locations in each vertebra rather than determining the L1 locations.

Although there is no direct comparison due to the lack of individual UVEL and LVEL analysis studies on each lumbar vertebra, we aim to compare our results with those of similar studies, such as Cobb angle. The variations in the angles of the UVEL and LVEL in the AP view of this study are comparable with Moftian et al. [30]. In their study, mean variations were 2.96° ± 2.13° and 2.18° ± 2.01° for manual labelling and computer-aided measurement system, respectively. Our measurements ranged from 0.577° ± 0.503° to 0.979° ± 0.871° (Table 4). Since the target task is different, it is hard to say that our result is superior than [30]. Cobb angle studies are conducted with whole spine images. Thoracic vertebrae generally have a larger labelling error than lumbar vertebrae. And the angle change in the lumbar spine is not as large as that in the thoracic spine. Therefore, the presented ICCs and RMSEs of the angles of the UVEL and LVEL in our study are limited to lumbar vertebrae analysis.

The main contribution and strength of this study is that it provides a reliability analysis of landmark point locations. With the remarkable development of artificial intelligence (AI), automatic X-ray image analysis algorithms have been actively studied in recent years [20,21]. There are several AI-based software programs, such as SpineAnalyzer (Optasia Medical, Cheadle Hulme, UK) [39] and PostureRay (PostureCo, Trinity, FL, USA) [40]. SpineAnalyzer is capable of automatically detecting six landmark points for each vertebra, while PostureRay detects four landmark points.

The performance of AI-based vertebral landmark point detection algorithms is evaluated by using the ground truth, which is typically the landmark point location labelled by a human expert. Therefore, it is important to establish reliable ground truth data. The labelled point location for the same vertebral image can vary among experts; therefore, high inter-rater reliability is required for the ground truth data.

Since no previous study analysed the distance errors of manually labelled landmark points among human experts in lumbar spine images, we aim to indirectly compare our results with a study on automatic landmark detection using AI. Yeh et al. [21] developed an AI algorithm to detect landmark point on whole spine LAT view images. In their results, Euclidean distance errors between human-labelled and AI-predicted locations ranged from 1.76 mm to 2.63 mm. These are comparable to our data, which reflect variations among human experts, ranging from 1.185 mm to 1.670 mm (Table 2). As described in the example above, the ground truth data that we provide can be utilized as a reference for evaluating AI algorithms.

Another strength of our study is the data size. A large set (1000 pairs) of AP and LAT view L-spine images were prepared, and 12 raters participated in the labelling process. Most previous studies used fewer than 100 images, and the number of raters varied from 2 to 10 [24,25,26,27,28,29,30,31]. To the best of our knowledge, our study uses the largest dataset among reliability studies of spinal landmarks to date. To address the large amount of data, the 12 raters were divided into two groups, and labelling was performed. As shown in Table 1 and Table 3, there were no significant differences between the two groups. We also double checked the possible differences between the two groups by employing 200 pairs of common data. All 12 raters labelled these common images, and the results are shown in Table 2 and Table 4. We effectively conducted a massive experiment with 1000 pairs of images and 12 raters, and this study will be a good reference for other studies.

Spinal alignment analysis using X-ray images has been mostly studied in Western medicine, such as orthopaedics and radiology. In our study, KMDs participated in the labelling process, and the definition of the landmark points was established based on Chuna manual medicine. While developing an AI algorithm using our data, we acknowledge the possibility that it may be limited to Chuna medicine. However, we anticipate that there will be no significant differences in locating the landmark points or drawing the UVELs and LVELs of the vertebrae across different medical practices. Further research is needed to investigate whether there are differences in landmark locations among other medical fields. If such differences are found to be significant, it might be necessary to develop specialized AI algorithms for each medical specialty.

## 5. Conclusions

This study presented the inter-rater reliability for the localization of four landmark points and endplate lines on AP and LAT views of L-spine X-ray images. An SOP was proposed by consensus of 12 manual medicine experts based on prior literature, and it helped to derive high reliability. A large data set of 2000 images was prepared for achieving high-quality experimental results. The ICCs for the landmark point locations and the angles of the vertebral endplate lines are presented. The resulting high ICCs verified the reliability of the labelling process using the proposed SOP. The mean and SD of each measurement were also presented, and they are expected to be used as a reference for the evaluation of AI-based landmark detection algorithms as well as manual labelling by experts. While this study used a large dataset of images, further research with an even greater number of cases could help to improve the development and evaluation of AI algorithms. In addition, future studies could compare the landmark points identified using Chuna manual medicine with those identified using morphometry in western medicine such as orthopaedics and radiology.

## Figures and Tables

**Table 1:** Mean variations and inter-rater reliability for landmark points according to rater group.

	AP					LAT				
	Group A		Group B			Group A		Group B		
	Mean (SD) ^†^	ICC (95% CI)	Mean (SD) ^†^	ICC (95% CI)	*p*-Value ^††^	Mean (SD) ^†^	ICC (95% CI)	Mean (SD) ^†^	ICC (95% CI)	*p*-Value ^††^
L1_p1	1.454 (0.898)	0.991 (0.990, 0.992)	1.397 (0.763)	0.992 (0.991, 0.993)	0.748	1.164 (0.737)	0.995 (0.994, 0.995)	1.047 (0.682)	0.996 (0.995, 0.996)	0.506
L1_p2	1.602 (1.060)	0.991 (0.989, 0.992)	1.538 (0.847)	0.991 (0.990, 0.992)	0.777	1.711 (1.079)	0.990 (0.988, 0.991)	1.487 (0.840)	0.993 (0.992, 0.993)	0.135
L1_p3	1.794 (1.163)	0.987 (0.985, 0.988)	1.798 (0.931)	0.988 (0.987, 0.989)	0.992	1.181 (0.717)	0.995 (0.994, 0.995)	1.042 (0.693)	0.996 (0.995, 0.996)	0.399
L1_p4	1.979 (1.294)	0.985 (0.983, 0.987)	1.787 (0.992)	0.988 (0.986, 0.989)	0.489	1.498 (0.972)	0.991 (0.990, 0.992)	1.350 (0.824)	0.993 (0.992, 0.994)	0.437
L2_p1	1.641 (0.992)	0.989 (0.988, 0.991)	1.667 (0.832)	0.990 (0.989, 0.991)	0.909	1.183 (0.744)	0.995 (0.994, 0.995)	1.129 (0.687)	0.995 (0.995, 0.996)	0.749
L2_p2	1.723 (1.091)	0.989 (0.988, 0.991)	1.748 (0.914)	0.989 (0.987, 0.990)	0.920	1.582 (0.968)	0.990 (0.989, 0.991)	1.504 (0.820)	0.991 (0.990, 0.992)	0.657
L2_p3	1.932 (1.209)	0.987 (0.985, 0.988)	1.997 (1.005)	0.987 (0.986, 0.988)	0.807	1.287 (0.772)	0.994 (0.993, 0.995)	1.127 (0.744)	0.995 (0.994, 0.995)	0.366
L2_p4	2.130 (1.354)	0.984 (0.982, 0.986)	1.985 (1.012)	0.988 (0.986, 0.989)	0.617	1.523 (0.953)	0.990 (0.989, 0.991)	1.342 (0.811)	0.993 (0.992, 0.994)	0.298
L3_p1	1.778 (1.063)	0.988 (0.987, 0.990)	1.814 (0.943)	0.989 (0.988, 0.990)	0.890	1.197 (0.751)	0.995 (0.994, 0.995)	1.228 (0.743)	0.994 (0.994, 0.995)	0.847
L3_p2	1.910 (1.199)	0.988 (0.986, 0.989)	1.835 (0.963)	0.989 (0.987, 0.990)	0.753	1.565 (0.988)	0.990 (0.988, 0.991)	1.500 (0.819)	0.991 (0.990, 0.992)	0.716
L3_p3	1.935 (1.224)	0.987 (0.985, 0.989)	2.054 (1.042)	0.988 (0.986, 0.989)	0.686	1.280 (0.786)	0.994 (0.993, 0.994)	1.112 (0.704)	0.995 (0.994, 0.996)	0.308
L3_p4	2.245 (1.437)	0.984 (0.982, 0.986)	2.141 (1.103)	0.986 (0.985, 0.988)	0.771	1.452 (0.926)	0.991 (0.990, 0.992)	1.347 (0.827)	0.992 (0.991, 0.993)	0.573
L4_p1	1.926 (1.190)	0.986 (0.984, 0.988)	1.928 (1.004)	0.988 (0.986, 0.989)	0.990	1.261 (0.846)	0.994 (0.993, 0.995)	1.329 (0.860)	0.992 (0.991, 0.993)	0.700
L4_p2	2.133 (1.368)	0.985 (0.983, 0.987)	2.000 (1.077)	0.986 (0.985, 0.988)	0.641	1.538 (0.973)	0.990 (0.989, 0.991)	1.473 (0.821)	0.991 (0.989, 0.992)	0.709
L4_p3	2.043 (1.276)	0.983 (0.981, 0.985)	2.066 (1.134)	0.986 (0.984, 0.988)	0.941	1.371 (0.880)	0.992 (0.991, 0.993)	1.165 (0.737)	0.994 (0.994, 0.995)	0.320
L4_p4	2.406 (1.519)	0.976 (0.973, 0.979)	2.144 (1.240)	0.985 (0.983, 0.986)	0.443	1.437 (0.894)	0.992 (0.991, 0.993)	1.336 (0.870)	0.992 (0.991, 0.993)	0.605
L5_p1	2.805 (1.914)	0.960 (0.954, 0.964)	2.492 (1.512)	0.970 (0.966, 0.974)	0.312	1.355 (1.085)	0.992 (0.991, 0.993)	1.534 (0.976)	0.990 (0.989, 0.991)	0.420
L5_p2	3.076 (2.116)	0.957 (0.951, 0.962)	2.530 (1.581)	0.970 (0.966, 0.973)	0.164	1.565 (1.086)	0.989 (0.987, 0.990)	1.478 (0.928)	0.990 (0.989, 0.991)	0.683
L5_p3	3.381 (2.279)	0.937 (0.929, 0.945)	3.036 (2.041)	0.948 (0.942, 0.954)	0.446	1.617 (1.451)	0.986 (0.984, 0.988)	1.320 (1.285)	0.989 (0.988, 0.991)	0.205
L5_p4	3.606 (2.485)	0.935 (0.926, 0.942)	2.918 (2.126)	0.947 (0.940, 0.953)	0.204	1.599 (1.279)	0.988 (0.986, 0.990)	1.520 (1.191)	0.988 (0.987, 0.990)	0.703

Abbreviations: AP, anteroposterior; LAT, lateral; SD, standard deviation; ICC, intra-class correlation; CI, confidence interval. ^†^ Unit: mm; ^††^ the *p*-values were obtained from *t*-statistics for testing the difference between two groups based on LMMs for each vertebra and landmark point.

**Table 2:** Mean variations and inter-rater reliability of landmark points assessed by all 12 raters.

	AP		LAT	
	Mean (SD) ^†^	ICC (95% CI)	Mean (SD) ^†^	ICC (95% CI)
L1_p1	1.469 (0.935)	0.991 (0.990, 0.993)	1.197 (0.822)	0.989 (0.986, 0.991)
L1_p2	1.563 (1.054)	0.991 (0.988, 0.992)	1.670 (1.065)	0.984 (0.981, 0.987)
L1_p3	1.782 (1.128)	0.988 (0.985, 0.990)	1.185 (0.808)	0.989 (0.986, 0.990)
L1_p4	1.883 (1.310)	0.986 (0.983, 0.989)	1.480 (1.017)	0.985 (0.981, 0.988)
L2_p1	1.674 (0.992)	0.990 (0.987, 0.991)	1.229 (0.810)	0.988 (0.985, 0.990)
L2_p2	1.702 (1.083)	0.990 (0.987, 0.992)	1.605 (1.014)	0.983 (0.979, 0.986)
L2_p3	1.919 (1.168)	0.987 (0.984, 0.989)	1.255 (0.808)	0.987 (0.984, 0.990)
L2_p4	2.008 (1.306)	0.987 (0.983, 0.989)	1.484 (1.005)	0.985 (0.982, 0.988)
L3_p1	1.774 (1.073)	0.989 (0.987, 0.991)	1.263 (0.830)	0.986 (0.983, 0.989)
L3_p2	1.801 (1.115)	0.990 (0.988, 0.992)	1.602 (1.033)	0.983 (0.979, 0.986)
L3_p3	1.933 (1.200)	0.989 (0.986, 0.991)	1.237 (0.835)	0.988 (0.985, 0.990)
L3_p4	2.107 (1.352)	0.987 (0.984, 0.989)	1.442 (0.982)	0.988 (0.985, 0.990)
L4_p1	1.902 (1.135)	0.988 (0.985, 0.990)	1.307 (0.865)	0.986 (0.983, 0.988)
L4_p2	1.986 (1.264)	0.987 (0.984, 0.990)	1.561 (0.997)	0.985 (0.981, 0.988)
L4_p3	2.037 (1.293)	0.985 (0.981, 0.988)	1.285 (0.884)	0.988 (0.984, 0.990)
L4_p4	2.236 (1.496)	0.981 (0.976, 0.984)	1.417 (0.952)	0.990 (0.987, 0.992)
L5_p1	2.677 (1.710)	0.966 (0.957, 0.972)	1.410 (0.924)	0.988 (0.985, 0.990)
L5_p2	2.722 (1.787)	0.967 (0.960, 0.972)	1.593 (1.073)	0.988 (0.985, 0.990)
L5_p3	3.330 (2.308)	0.937 (0.922, 0.947)	1.521 (1.741)	0.984 (0.981, 0.987)
L5_p4	3.430 (2.450)	0.934 (0.920, 0.946)	1.645 (1.480)	0.988 (0.985, 0.990)

Abbreviations: AP, anteroposterior; LAT, lateral; SD, standard deviation; ICC, intra-class correlation; CI, confidence interval. ^†^ Unit: mm.

**Table 3:** Mean variations and inter-rater reliability of the angles of the UVEL and LVEL according to rater group.

	AP					LAT				
	Group A		Group B			Group A		Group B		
	Mean (SD) ^†^	ICC (95% CI)	Mean (SD) ^†^	ICC (95% CI)	*p*-Value ^††^	Mean (SD) ^†^	ICC (95% CI)	Mean (SD) ^†^	ICC (95% CI)	*p*-Value ^††^
L1_UVEL	0.716 (0.603)	0.921 (0.909, 0.930)	0.678 (0.566)	0.907 (0.895, 0.917)	0.664	1.170 (0.944)	0.938 (0.930, 0.945)	1.162 (0.940)	0.938 (0.922, 0.950)	0.958
L1_LVEL	0.797 (0.660)	0.909 (0.896, 0.919)	0.723 (0.617)	0.901 (0.886, 0.914)	0.419	1.054 (0.894)	0.947 (0.934, 0.955)	0.978 (0.813)	0.952 (0.938, 0.961)	0.618
L2_UVEL	0.735 (0.614)	0.917 (0.905, 0.928)	0.677 (0.570)	0.909 (0.896, 0.920)	0.583	1.089 (0.877)	0.942 (0.933, 0.949)	1.078 (0.873)	0.940 (0.921, 0.952)	0.949
L2_LVEL	0.764 (0.650)	0.915 (0.905, 0.926)	0.664 (0.560)	0.921 (0.908, 0.930)	0.303	0.942 (0.787)	0.959 (0.952, 0.964)	0.982 (0.812)	0.952 (0.941, 0.959)	0.732
L3_UVEL	0.669 (0.604)	0.925 (0.913, 0.935)	0.613 (0.530)	0.930 (0.922, 0.938)	0.572	1.024 (0.837)	0.952 (0.945, 0.958)	1.088 (0.862)	0.944 (0.933, 0.952)	0.623
L3_LVEL	0.635 (0.573)	0.939 (0.931, 0.945)	0.572 (0.498)	0.942 (0.933, 0.949)	0.543	0.918 (0.786)	0.967 (0.963, 0.971)	0.982 (0.807)	0.960 (0.949, 0.967)	0.544
L4_UVEL	0.686 (0.600)	0.923 (0.913, 0.933)	0.621 (0.560)	0.926 (0.916, 0.934)	0.562	1.024 (0.852)	0.963 (0.957, 0.967)	1.149 (0.928)	0.947 (0.937, 0.955)	0.343
L4_LVEL	0.754 (0.682)	0.905 (0.892, 0.915)	0.662 (0.611)	0.915 (0.894, 0.927)	0.433	1.062 (0.884)	0.969 (0.965, 0.972)	1.097 (0.915)	0.961 (0.952, 0.967)	0.794
L5_UVEL	0.950 (0.828)	0.817 (0.796, 0.835)	0.877 (0.790)	0.827 (0.800, 0.849)	0.499	1.229 (1.029)	0.962 (0.955, 0.967)	1.319 (1.102)	0.951 (0.941, 0.957)	0.561
L5_LVEL	1.042 (0.913)	0.777 (0.755, 0.799)	1.033 (0.909)	0.759 (0.708, 0.793)	0.933	1.451 (1.322)	0.950 (0.943, 0.956)	1.532 (1.401)	0.939 (0.924, 0.949)	0.702

Abbreviations: AP, anteroposterior; LAT, lateral; SD, standard deviation; ICC, intra-class correlation; CI, confidence interval; UVEL, upper vertebral endplate line; LVEL, lower vertebral endplate line. ^†^ Unit: degree; ^††^ the *p*-values were obtained from t-statistics for testing the difference between two groups based on LMMs for each vertebra and landmark point.

**Table 4:** Mean variations and inter-rater reliability of the angles of the UVEL and LVEL assessed by all 12 raters.

	AP		LAT	
	Mean (SD) ^†^	ICC (95% CI)	Mean (SD) ^†^	ICC (95% CI)
L1_UVEL	0.661 (0.548)	0.931 (0.916, 0.943)	1.141 (0.953)	0.944 (0.928, 0.956)
L1_LVEL	0.718 (0.614)	0.918 (0.899, 0.931)	1.055 (0.916)	0.948 (0.930, 0.960)
L2_UVEL	0.678 (0.566)	0.921 (0.903, 0.935)	1.049 (0.887)	0.946 (0.928, 0.958)
L2_LVEL	0.677 (0.573)	0.926 (0.909, 0.939)	0.936 (0.748)	0.957 (0.947, 0.965)
L3_UVEL	0.606 (0.525)	0.933 (0.918, 0.945)	1.066 (0.875)	0.942 (0.928, 0.954)
L3_LVEL	0.577 (0.503)	0.942 (0.928, 0.952)	0.906 (0.787)	0.963 (0.953, 0.970)
L4_UVEL	0.643 (0.572)	0.933 (0.917, 0.943)	1.067 (0.893)	0.953 (0.941, 0.960)
L4_LVEL	0.689 (0.640)	0.924 (0.907, 0.938)	1.037 (0.862)	0.960 (0.951, 0.968)
L5_UVEL	0.901 (0.791)	0.839 (0.801, 0.865)	1.240 (1.007)	0.957 (0.947, 0.964)
L5_LVEL	0.979 (0.871)	0.801 (0.764, 0.834)	1.486 (1.448)	0.942 (0.929, 0.952)

Abbreviations: AP, anteroposterior; LAT, lateral; SD, standard deviation; ICC, intra-class correlation; CI, confidence interval. ^†^ Unit: degree.

## Data Availability

The datasets used and analysed during the current study are available from the corresponding author on reasonable request.

## Supplementary Materials

The following supporting information can be downloaded at: https://www.mdpi.com/article/10.3390/diagnostics13081411/s1, Table S1: Mean variations and inter-rater reliability of landmark points in the x and y directions according to rater groups (AP), Table S2: Mean variations and inter-rater reliability of landmark points in the x and y directions according to rater groups (LAT), Table S3: Mean variations and inter-rater reliability of each point in the x and y directions assessed by all 12 raters, Figure S1: Boxplots of Euclidean distances from the mean location of all 12 raters’ labelled landmark points on the AP view, Figure S2: Boxplots of Euclidean distances from the mean location of all 12 raters’ labelled landmark points on the LAT view, Figure S3: Boxplots of the RMSE of the angles of the UVEL and LVEL for all 12 raters, Table S4: Summary of the omnibus test result for the effect of age and sex on the mean variations of all 12 raters’ labelled landmark points using the LMM, Table S5: Summary of the omnibus test result for the effect of age and sex on the mean variations of angles of the UVEL and LVEL using the LMM.
