# Supplementary material for: Reliability Analysis of Vertebral Landmark Labelling on Lumbar Spine X-ray Images
Source: Diagnostics (Basel). 2023 Apr 13;13(8):1411. doi: 10.3390/diagnostics13081411 (PMC10137367; doi:10.3390/diagnostics13081411)
Supplement: Supplementary file 1 [file diagnostics-13-01411-s001.zip › diagnostics-2200628-supplementary.pdf]

Supplementary Materials

Reliability Analysis of Vertebral Landmark Labelling on Lumbar Spine X-ray Images

Table S1. Mean variations and inter-rater reliability of landmark points in the x and y directions according to rater groups (AP).

	Group A				Group B			
	Mean (SD)†		ICC (95% CI)		Mean (SD)†		ICC (95% CI)	
	x	y	x	y	x	y	x	y
L1_p1	1.021 (0.955)	0.800 (0.659)	0.972 (0.962, 0.980)	0.998 (0.996, 0.998)	1.071 (0.750)	0.741 (0.620)	0.977 (0.959, 0.987)	0.998 (0.997, 0.998)
L1_p2	1.243 (1.109)	0.746 (0.663)	0.959 (0.942, 0.968)	0.998 (0.997, 0.998)	1.186 (0.871)	0.774 (0.634)	0.969 (0.945, 0.982)	0.998 (0.997, 0.998)
L1_p3	1.306 (1.171)	0.963 (0.832)	0.952 (0.927, 0.966)	0.996 (0.995, 0.997)	1.528 (0.906)	0.764 (0.695)	0.955 (0.912, 0.978)	0.998 (0.997, 0.998)
L1_p4	1.428 (1.341)	1.076 (0.851)	0.937 (0.919, 0.951)	0.996 (0.993, 0.997)	1.462 (0.997)	0.815 (0.713)	0.951 (0.912, 0.974)	0.997 (0.997, 0.998)
L2_p1	1.197 (1.027)	0.885 (0.717)	0.961 (0.943, 0.971)	0.997 (0.995, 0.998)	1.326 (0.843)	0.821 (0.661)	0.964 (0.938, 0.979)	0.997 (0.996, 0.998)
L2_p2	1.351 (1.151)	0.802 (0.678)	0.948 (0.932, 0.958)	0.997 (0.996, 0.998)	1.372 (0.943)	0.866 (0.678)	0.956 (0.925, 0.975)	0.997 (0.996, 0.998)
L2_p3	1.491 (1.257)	0.939 (0.797)	0.938 (0.901, 0.958)	0.996 (0.994, 0.997)	1.744 (1.034)	0.777 (0.637)	0.938 (0.881, 0.969)	0.998 (0.997, 0.998)
L2_p4	1.624 (1.432)	1.062 (0.825)	0.922 (0.886, 0.942)	0.996 (0.993, 0.997)	1.686 (1.090)	0.811 (0.634)	0.933 (0.876, 0.966)	0.998 (0.996, 0.998)
L3_p1	1.382 (1.137)	0.867 (0.679)	0.947 (0.921, 0.962)	0.997 (0.995, 0.998)	1.519 (1.010)	0.778 (0.611)	0.948 (0.906, 0.970)	0.998 (0.997, 0.998)
L3_p2	1.582 (1.288)	0.778 (0.641)	0.932 (0.904, 0.948)	0.997 (0.996, 0.998)	1.548 (1.026)	0.781 (0.575)	0.941 (0.892, 0.966)	0.998 (0.996, 0.998)
L3_p3	1.571 (1.314)	0.827 (0.704)	0.931 (0.891, 0.955)	0.997 (0.995, 0.998)	1.842 (1.110)	0.708 (0.555)	0.926 (0.865, 0.963)	0.998 (0.997, 0.999)
L3_p4	1.844 (1.545)	0.927 (0.756)	0.910 (0.868, 0.939)	0.997 (0.994, 0.998)	1.898 (1.196)	0.740 (0.577)	0.915 (0.846, 0.957)	0.998 (0.997, 0.999)
L4_p1	1.499 (1.244)	0.941 (0.756)	0.937 (0.911, 0.955)	0.996 (0.994, 0.998)	1.569 (1.071)	0.872 (0.690)	0.940 (0.895, 0.967)	0.997 (0.995, 0.998)
L4_p2	1.794 (1.481)	0.819 (0.677)	0.918 (0.882, 0.938)	0.997 (0.996, 0.998)	1.674 (1.139)	0.858 (0.664)	0.930 (0.876, 0.962)	0.997 (0.996, 0.998)
L4_p3	1.584 (1.311)	0.979 (0.874)	0.933 (0.899, 0.955)	0.996 (0.994, 0.997)	1.802 (1.151)	0.773 (0.730)	0.925 (0.859, 0.960)	0.997 (0.997, 0.998)
L4_p4	1.869 (1.583)	1.138 (0.979)	0.914 (0.874, 0.938)	0.994 (0.992, 0.996)	1.838 (1.255)	0.821 (0.811)	0.917 (0.857, 0.959)	0.997 (0.996, 0.998)
L5_p1	1.858 (1.589)	1.694 (1.704)	0.907 (0.860, 0.933)	0.985 (0.978, 0.989)	1.701 (1.256)	1.496 (1.418)	0.925 (0.888, 0.946)	0.990 (0.986, 0.992)
L5_p2	2.184 (1.845)	1.703 (1.763)	0.881 (0.833, 0.915)	0.985 (0.978, 0.988)	1.776 (1.337)	1.483 (1.418)	0.916 (0.876, 0.940)	0.990 (0.987, 0.992)
L5_p3	2.008 (1.645)	2.291 (2.230)	0.904 (0.857, 0.933)	0.974 (0.963, 0.979)	1.978 (1.428)	1.952 (1.988)	0.907 (0.851, 0.946)	0.982 (0.974, 0.986)
L5_p4	2.290 (1.964)	2.309 (2.264)	0.877 (0.818, 0.916)	0.974 (0.964, 0.979)	1.745 (1.464)	2.004 (2.053)	0.917 (0.875, 0.940)	0.980 (0.972, 0.985)

Abbreviation: SD, standard deviation; ICC, intra-class correlation; CI, confidence interval.
†Unit: mm.

Table S2. Mean variations and inter-rater reliability of landmark points in the x and y directions according to rater groups (LAT).

	Group A				Group B			
	Mean (SD)†		ICC (95% CI)		Mean (SD)†		ICC (95% CI)	
	x	y	x	y	x	y	x	y
L1_p1	0.795 (0.767)	0.706 (0.731)	0.995 (0.993, 0.996)	0.999 (0.998, 0.999)	0.786 (0.664)	0.614 (0.810)	0.996 (0.995, 0.997)	0.999 (0.998, 0.999)
L1_p2	1.255 (1.129)	0.936 (0.839)	0.988 (0.982, 0.991)	0.998 (0.996, 0.998)	1.021 (0.860)	0.905 (0.907)	0.993 (0.991, 0.995)	0.998 (0.996, 0.999)
L1_p3	0.923 (0.790)	0.603 (0.680)	0.993 (0.990, 0.995)	0.999 (0.999, 0.999)	0.839 (0.700)	0.564 (0.789)	0.995 (0.993, 0.996)	0.999 (0.999, 0.999)
L1_p4	1.064 (0.959)	0.860 (0.869)	0.990 (0.987, 0.992)	0.998 (0.997, 0.998)	1.040 (0.848)	0.731 (0.834)	0.992 (0.987, 0.995)	0.998 (0.998, 0.999)
L2_p1	0.876 (0.800)	0.659 (0.733)	0.993 (0.990, 0.994)	0.999 (0.998, 0.999)	0.843 (0.691)	0.664 (0.866)	0.995 (0.992, 0.996)	0.999 (0.998, 0.999)
L2_p2	1.149 (1.036)	0.883 (0.828)	0.988 (0.983, 0.991)	0.998 (0.997, 0.998)	1.032 (0.843)	0.939 (0.948)	0.993 (0.989, 0.995)	0.997 (0.995, 0.999)
L2_p3	0.987 (0.833)	0.683 (0.780)	0.990 (0.986, 0.993)	0.999 (0.998, 0.999)	0.901 (0.737)	0.605 (0.915)	0.994 (0.990, 0.996)	0.999 (0.999, 0.999)
L2_p4	1.112 (0.986)	0.834 (0.842)	0.987 (0.983, 0.990)	0.998 (0.997, 0.998)	1.050 (0.864)	0.693 (0.840)	0.991 (0.985, 0.995)	0.998 (0.998, 0.999)
L3_p1	0.911 (0.813)	0.645 (0.821)	0.991 (0.988, 0.993)	0.999 (0.998, 0.999)	0.905 (0.737)	0.726 (1.031)	0.993 (0.991, 0.995)	0.998 (0.997, 0.999)
L3_p2	1.157 (1.034)	0.850 (0.847)	0.986 (0.981, 0.989)	0.998 (0.997, 0.998)	1.021 (0.846)	0.935 (0.962)	0.992 (0.987, 0.994)	0.997 (0.995, 0.998)
L3_p3	0.921 (0.804)	0.748 (0.851)	0.990 (0.986, 0.993)	0.998 (0.998, 0.999)	0.881 (0.706)	0.605 (0.945)	0.993 (0.990, 0.995)	0.999 (0.999, 0.999)
L3_p4	1.100 (0.971)	0.748 (0.785)	0.986 (0.982, 0.989)	0.998 (0.997, 0.998)	1.057 (0.866)	0.689 (0.838)	0.991 (0.984, 0.994)	0.998 (0.997, 0.999)
L4_p1	0.965 (0.902)	0.675 (0.874)	0.989 (0.986, 0.991)	0.999 (0.998, 0.999)	0.935 (0.854)	0.830 (1.106)	0.992 (0.989, 0.994)	0.998 (0.997, 0.998)
L4_p2	1.127 (1.007)	0.859 (0.846)	0.985 (0.980, 0.988)	0.997 (0.997, 0.998)	0.999 (0.822)	0.935 (0.966)	0.992 (0.988, 0.994)	0.997 (0.995, 0.998)
L4_p3	0.926 (0.886)	0.855 (0.952)	0.991 (0.988, 0.992)	0.998 (0.997, 0.998)	0.907 (0.789)	0.654 (0.997)	0.994 (0.990, 0.995)	0.999 (0.998, 0.999)
L4_p4	1.113 (0.966)	0.717 (0.727)	0.987 (0.983, 0.990)	0.998 (0.998, 0.998)	1.047 (0.890)	0.707 (0.816)	0.991 (0.985, 0.995)	0.998 (0.997, 0.998)
L5_p1	0.982 (1.080)	0.805 (0.895)	0.990 (0.987, 0.992)	0.997 (0.997, 0.998)	1.013 (1.019)	1.032 (1.007)	0.993 (0.989, 0.995)	0.996 (0.994, 0.998)
L5_p2	1.110 (1.089)	0.910 (0.843)	0.987 (0.983, 0.990)	0.997 (0.996, 0.997)	0.988 (0.914)	0.965 (0.874)	0.993 (0.990, 0.994)	0.996 (0.994, 0.998)
L5_p3	0.962 (1.224)	1.094 (1.163)	0.990 (0.988, 0.991)	0.995 (0.993, 0.996)	0.921 (1.153)	0.815 (0.896)	0.992 (0.990, 0.993)	0.997 (0.996, 0.997)
L5_p4	1.159 (1.216)	0.883 (0.893)	0.987 (0.982, 0.989)	0.997 (0.996, 0.997)	1.076 (1.083)	0.900 (0.891)	0.991 (0.986, 0.993)	0.996 (0.996, 0.997)

Abbreviation: SD, standard deviation; ICC, intra-class correlation; CI, confidence interval.
†Unit: mm.

Table S3. Mean variations and inter-rater reliability of each point in the x and y directions assessed by all 12 raters.

	AP				LAT			
	Mean (SD) †		ICC (95% CI)		Mean (SD) †		ICC (95% CI)	
	x	y	x	y	x	y	x	y
L1_p1	1.110 (1.020)	0.789 (0.685)	0.967 (0.952, 0.978)	0.998 (0.997, 0.998)	0.916 (0.882)	0.738 (1.126)	0.993 (0.990, 0.994)	0.997 (0.996, 0.998)
L1_p2	1.187 (1.122)	0.803 (0.699)	0.958 (0.942, 0.969)	0.998 (0.997, 0.998)	1.212 (1.151)	1.005 (1.178)	0.988 (0.983, 0.991)	0.996 (0.995, 0.997)
L1_p3	1.409 (1.191)	0.894 (0.788)	0.946 (0.923, 0.964)	0.997 (0.996, 0.998)	1.015 (0.913)	0.653 (1.099)	0.990 (0.986, 0.993)	0.997 (0.996, 0.998)
L1_p4	1.438 (1.379)	0.986 (0.837)	0.932 (0.903, 0.950)	0.997 (0.995, 0.998)	1.098 (1.034)	0.909 (1.206)	0.989 (0.984, 0.991)	0.996 (0.995, 0.997)
L2_p1	1.324 (1.084)	0.840 (0.693)	0.953 (0.932, 0.968)	0.998 (0.997, 0.998)	1.002 (0.925)	0.718 (1.177)	0.990 (0.985, 0.993)	0.997 (0.996, 0.997)
L2_p2	1.340 (1.168)	0.844 (0.693)	0.944 (0.923, 0.958)	0.998 (0.997, 0.998)	1.167 (1.121)	1.000 (1.243)	0.987 (0.981, 0.990)	0.995 (0.993, 0.997)
L2_p3	1.545 (1.263)	0.905 (0.785)	0.934 (0.901, 0.957)	0.997 (0.996, 0.998)	1.066 (0.929)	0.690 (1.261)	0.988 (0.983, 0.992)	0.996 (0.995, 0.997)
L2_p4	1.595 (1.415)	0.970 (0.797)	0.919 (0.885, 0.943)	0.997 (0.995, 0.998)	1.154 (1.095)	0.829 (1.201)	0.986 (0.980, 0.990)	0.996 (0.995, 0.997)
L3_p1	1.479 (1.212)	0.784 (0.633)	0.940 (0.915, 0.958)	0.998 (0.997, 0.999)	1.032 (0.933)	0.736 (1.422)	0.988 (0.983, 0.991)	0.995 (0.994, 0.996)
L3_p2	1.510 (1.235)	0.766 (0.607)	0.932 (0.903, 0.951)	0.998 (0.997, 0.999)	1.167 (1.123)	0.976 (1.284)	0.985 (0.979, 0.989)	0.995 (0.993, 0.996)
L3_p3	1.652 (1.341)	0.776 (0.666)	0.926 (0.890, 0.950)	0.998 (0.997, 0.999)	0.990 (0.901)	0.742 (1.367)	0.988 (0.983, 0.991)	0.996 (0.994, 0.996)
L3_p4	1.778 (1.473)	0.870 (0.719)	0.910 (0.870, 0.938)	0.997 (0.996, 0.998)	1.144 (1.080)	0.762 (1.161)	0.985 (0.979, 0.989)	0.996 (0.995, 0.997)
L4_p1	1.543 (1.231)	0.897 (0.756)	0.933 (0.900, 0.954)	0.997 (0.996, 0.998)	1.022 (0.968)	0.825 (1.516)	0.987 (0.982, 0.990)	0.995 (0.993, 0.996)
L4_p2	1.648 (1.412)	0.836 (0.701)	0.920 (0.888, 0.942)	0.998 (0.997, 0.998)	1.127 (1.062)	0.987 (1.286)	0.985 (0.980, 0.989)	0.995 (0.993, 0.996)
L4_p3	1.662 (1.366)	0.919 (0.890)	0.924 (0.886, 0.946)	0.997 (0.995, 0.997)	0.980 (1.014)	0.834 (1.461)	0.988 (0.982, 0.991)	0.995 (0.994, 0.996)
L4_p4	1.772 (1.564)	1.058 (0.996)	0.910 (0.870, 0.936)	0.996 (0.994, 0.997)	1.139 (1.070)	0.771 (1.083)	0.986 (0.979, 0.989)	0.996 (0.995, 0.997)
L5_p1	1.811 (1.471)	1.647 (1.608)	0.911 (0.875, 0.934)	0.989 (0.984, 0.992)	1.063 (1.249)	0.950 (1.166)	0.986 (0.981, 0.989)	0.996 (0.995, 0.997)
L5_p2	1.956 (1.647)	1.564 (1.537)	0.894 (0.851, 0.919)	0.990 (0.986, 0.992)	1.142 (1.183)	1.038 (1.046)	0.986 (0.982, 0.989)	0.996 (0.994, 0.997)
L5_p3	1.948 (1.575)	2.319 (2.399)	0.907 (0.867, 0.937)	0.976 (0.967, 0.982)	1.015 (1.578)	1.033 (1.231)	0.984 (0.980, 0.987)	0.995 (0.993, 0.997)
L5_p4	2.039 (1.817)	2.368 (2.416)	0.887 (0.845, 0.919)	0.975 (0.967, 0.981)	1.238 (1.452)	0.943 (0.995)	0.983 (0.977, 0.987)	0.996 (0.995, 0.997)

Abbreviation: AP, anteroposterior; LAT, lateral; SD, standard deviation; ICC, intra-class correlation; CI, confidence interval.

†Unit: mm.

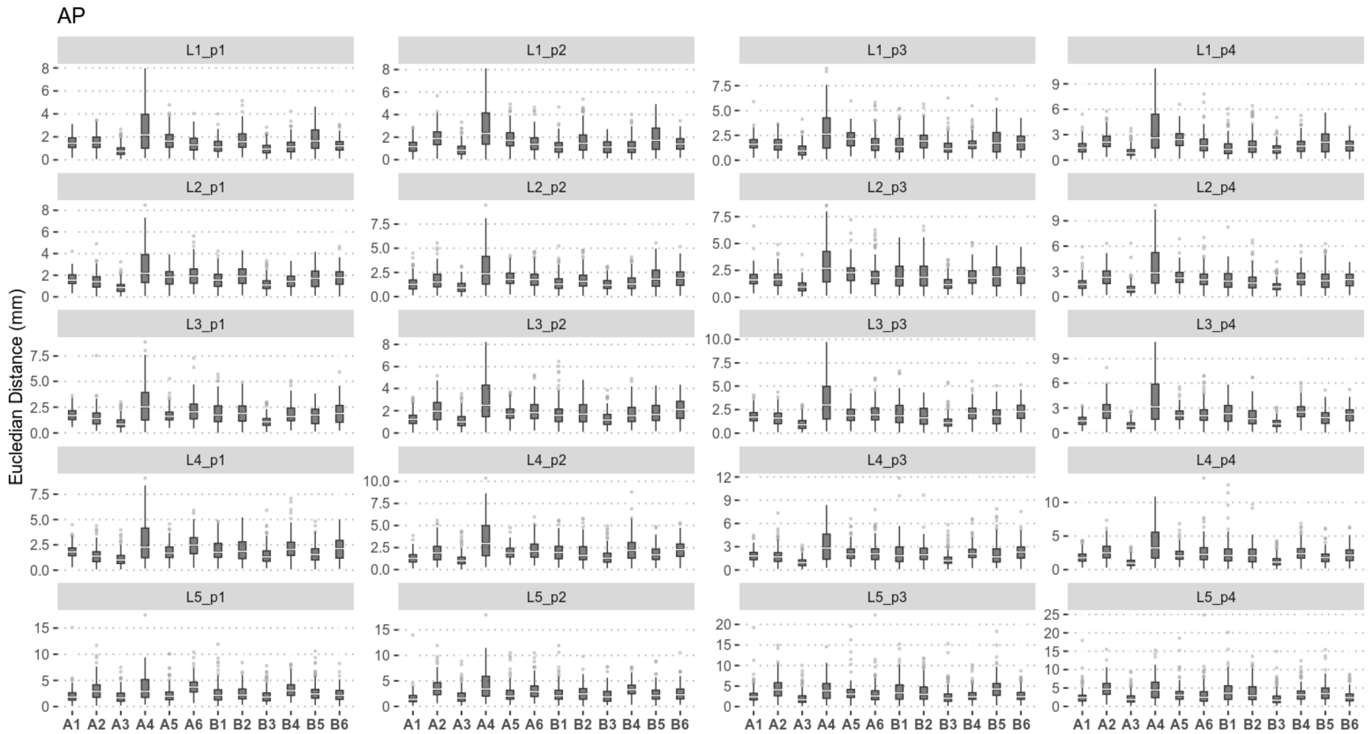

Figure S1. Boxplots of Euclidean distances from the mean location of all 12 raters' labelled landmark points on the AP view. The x-axis of each panel represents the identifier of raters within the group A and B.

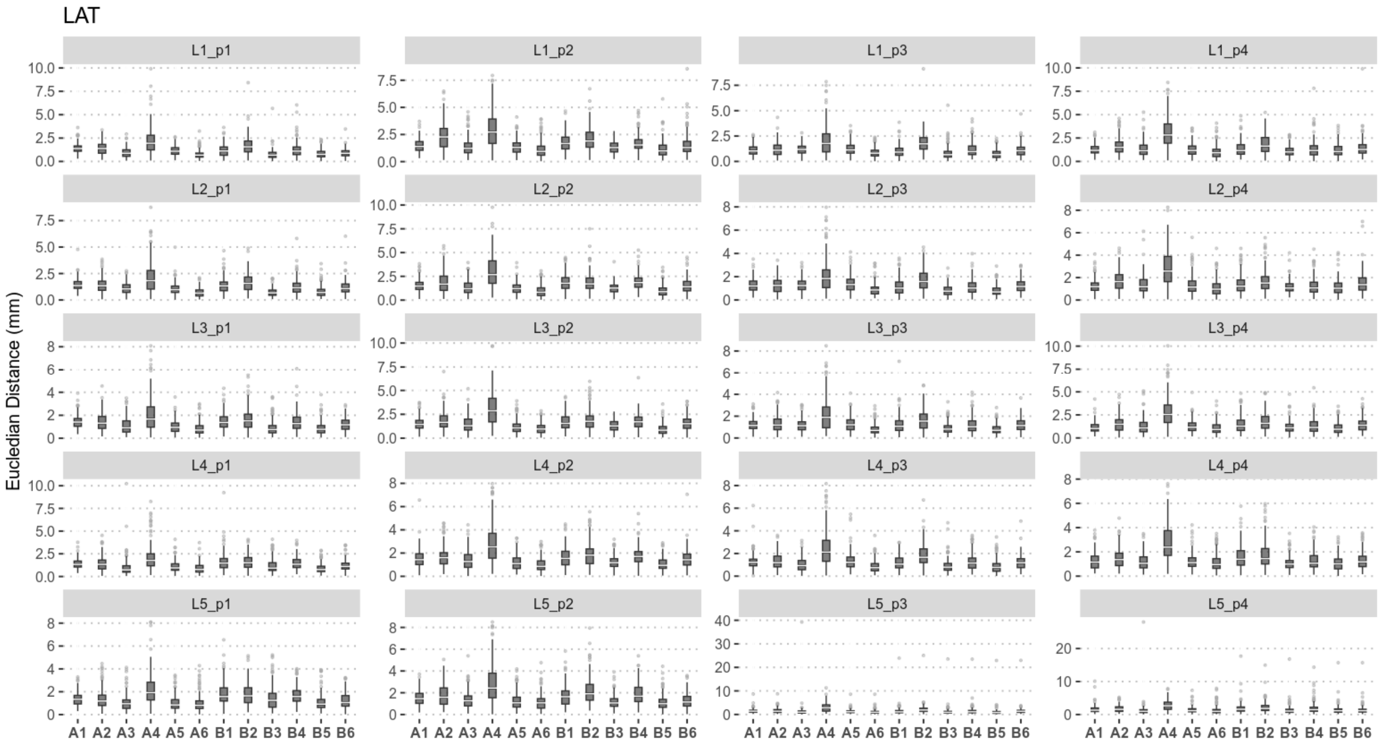

Figure S2. Boxplots of Euclidean distances from the mean location of all 12 raters' labelled landmark points on the LAT view. The x-axis of each panel represents the identifier of raters within the group A and B.

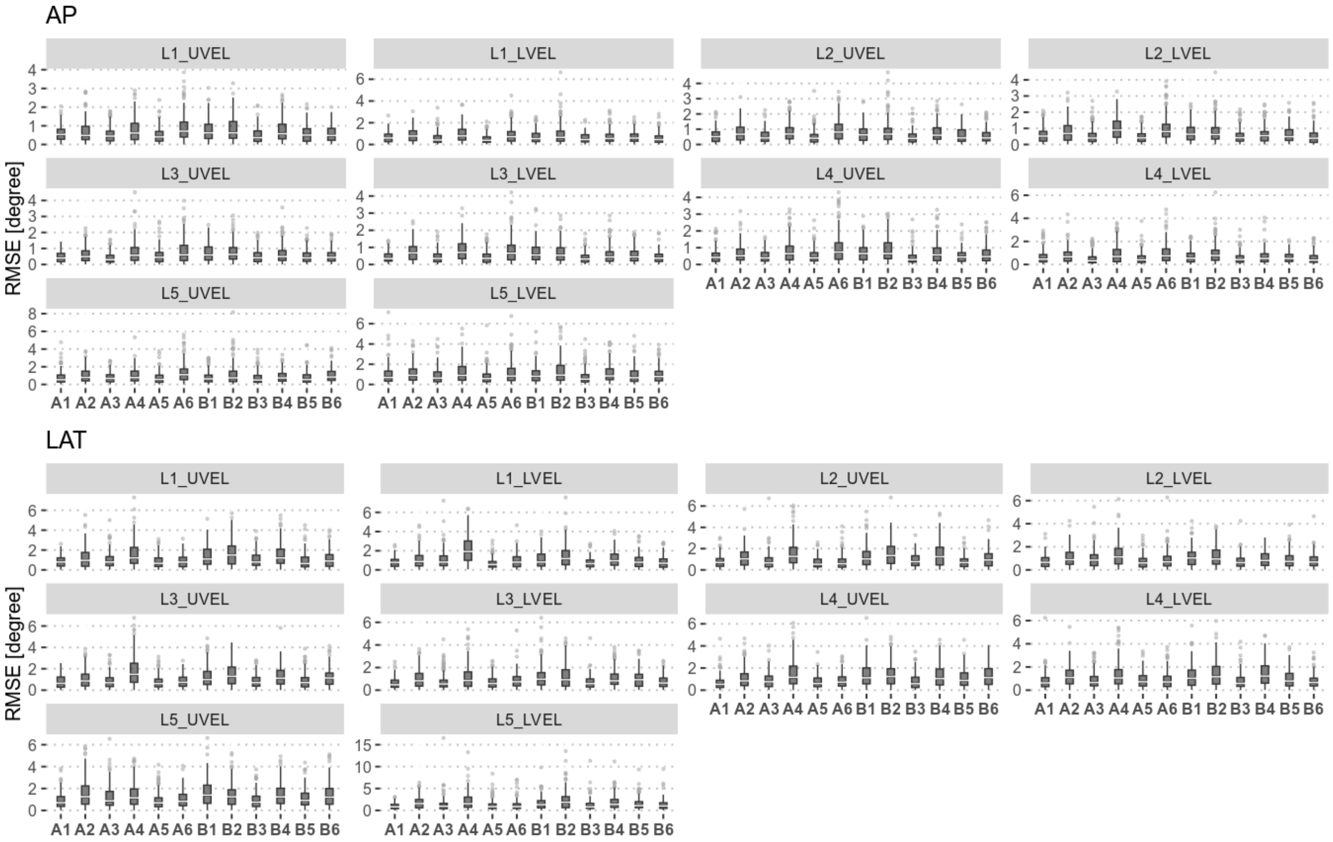

Figure S3. Boxplots of the RMSE of the angles of the UVEL and LVEL for all 12 raters. The x-axis of each panel represents the identifier of raters within the group A and B. Abbreviation: AP, antero-posterior; LAT, lateral.

Table S4. Summary of the omnibus test result for the effect of age and sex on the mean variations of all 12 raters' labelled landmark points using the LMM.

	AP			LAT		
	Age	Sex	Age×Sex	Age	Sex	Age×Sex
L1_p1	0.0011	0.0693	0.9070	0.2891	0.0496	0.7155
L1_p2	2.0.E-05	0.0006	0.8919	0.1283	0.0703	0.7952
L1_p3	0.0028	1.2.E-05	0.2053	0.5323	0.0005	0.4145
L1_p4	6.9.E-06	0.0005	0.0536	0.8876	0.7085	0.8966
L2_p1	0.0005	0.0004	0.1830	0.3759	0.0011	0.5325
L2_p2	0.0001	0.0128	0.0664	0.9669	0.4908	0.4682
L2_p3	0.0120	0.0080	0.7550	0.6614	0.0121	0.7648
L2_p4	0.0009	0.0138	0.2753	0.8639	0.4726	0.9473
L3_p1	0.2877	0.0007	0.2495	0.0064	0.0001	0.9188
L3_p2	0.2519	0.0037	0.2764	0.8985	0.9405	0.9563
L3_p3	0.1119	0.0026	0.1095	0.4108	0.0006	0.7692
L3_p4	0.0294	0.0005	0.3783	0.4339	0.0763	0.8376
L4_p1	0.0059	0.0963	0.4661	0.0711	0.0252	0.6861
L4_p2	0.2543	0.0055	0.2989	0.5477	0.0393	0.4331
L4_p3	0.0057	0.3263	0.4357	0.4257	0.0412	0.9713
L4_p4	0.0057	0.8249	0.8546	0.1772	0.0026	0.8282
L5_p1	0.0065	0.1737	0.9425	0.0044	0.0296	0.4322
L5_p2	0.0003	0.2069	0.6788	0.0479	0.0742	0.5127
L5_p3	0.0065	0.7951	0.5903	0.1539	0.3719	0.5908
L5_p4	0.0031	0.7363	0.5982	0.1616	0.6823	0.4345

Values represent p-values for age, sex and interaction between both fixed factors. The continuous variable age was grouped into four categories; less than 40, 40 to 54, 55 to 64 and greater than 65.

Table S5. Summary of the omnibus test result for the effect of age and sex on the mean variations of angles of the UVEL and LVEL using the LMM.

	AP			LAT		
	Age	Sex	Age×Sex	Age	Sex	Age×Sex
L1_UVEL	0.6957	0.0202	0.0161	0.0084	0.2860	0.4669
L1_LVEL	0.0161	0.3090	0.6226	0.2455	0.8094	0.2441
L2_UVEL	0.1240	0.5169	0.5149	0.2097	0.0973	0.9381
L2_LVEL	0.0121	0.1063	0.4469	0.3268	0.0602	0.4581
L3_UVEL	0.0734	0.0675	0.0020	0.0933	0.5041	0.3100
L3_LVEL	0.0998	0.0600	0.0359	0.2715	0.2273	0.0651
L4_UVEL	0.0698	0.0029	0.4008	0.1112	0.2299	0.6454
L4_LVEL	0.0136	0.1533	0.8312	0.7274	0.6538	0.7845
L5_UVEL	0.0039	0.2200	0.7164	0.0589	0.9988	0.0172
L5_LVEL	0.0006	0.4125	0.2684	0.0540	0.9603	0.5025

Values represent p-values for age, sex and interaction between both fixed factors. The continuous variable age was grouped into four categories; less than 40, 40 to 54, 55 to 64 and greater than 65.
